# What are the risk factors for HIV in men who have sex with men in Ho Chi Minh City, Vietnam?- A cross-sectional study

**DOI:** 10.1186/s12889-016-3088-8

**Published:** 2016-05-16

**Authors:** Thi My Dung Le, Patricia C. Lee, Donald E. Stewart, Thanh Nguyen Long, Cuong Nguyen Quoc

**Affiliations:** Care & Treatment Division, The Global Fund Supported Project on HIV/AIDS, Ministry of Health, Level 8, No.14 Lang Ha street, Ba Dinh district, Hanoi, Vietnam; Menzies Health Institute Queensland, Southport, Queensland Australia; School of Medicine, Griffith University Gold Coast campus, Parklands Drive, Southport, Queensland 4222 Australia; School of Medicine, Griffith University South Bank campus, 226 Grey Street, South Bank, Queensland 4101 Australia; Ministry of Health, 138 GiangVo Street, Ba Dinh District, Hanoi, Vietnam; Family of Health International 360, Vietnam country office, No. 8 Ly Thuong Kiet, Phan Chu Trinh ward, Hoan Kiem District, Hanoi, Vietnam

**Keywords:** HIV, Men who have sex with men (MSM), Ho Chi Minh City (HCMC), Risk factors

## Abstract

**Background:**

The number of people living with HIV (PLWH) in Vietnam was estimated to rise from 156,802 in 2009 to 256,000 in 2014. Although the number of new HIV reported cases has decreased by roughly 14,000 cases per year from 2010 to 2013 a concerning increase in HIV prevalence has been identified among men who have sex with men (MSM) from 1.7 % in 2005 to 2.4 % in 2013. There are signs of increased HIV (+) prevalence among MSM in a number of cities/provinces, especially in the two largest cities, Ho Chi Minh City (HCMC) and Hanoi. HCMC is the country’s major “hot spot” for HIV/AIDS, with over a third of the total national AIDS patients. This paper is based on a secondary analysis of Integrated Biological and Behavioural Surveillance (IBBS) data collected in Vietnam in 2009 to examine the research question “Do behavioural risk factors contribute to HIV infection among the MSM population in HCMC?”.

**Methods:**

A cross-sectional design was employed to sample males aged over 15 from communities in HCMC, who reported having any types of sex with another man at least once during the last 12 months. Participants (399) were recruited using the respondent driven sampling (RDS) method and provided both biological data (specimens) and behavioural data collected through a questionnaire survey.

**Results:**

The study found high HIV prevalence (14.8 %) among the MSM sample from HCMC. Multivariate analysis found age and level of formal education completed, to be significantly associated with HIV infection. MSM aged over 25 were more likely to be HIV (+) than the younger group (OR = 7.82, 95 %CI = 3.37–18.16, *p* < 0.001); as were participants who had low educational (OR = 2.74, *p* < 0.05) and medium educational levels (OR = 2.68, *p* < 0.05). In addition, those participants who had anal sex with male partners (OR = 2.7, *p* < 0.05) and whose sexual partners injected drugs (OR = 2.24, *p* < 0.05) and who felt at risk of HIV infection (OR = 2.42, *p* < 0.01) had a higher risk of HIV infection.

**Conclusions:**

The high proportion of HIV (+) MSM in our sample from HCMC indicates that we need a better understanding of MSM behaviour patterns, risk practices and social networks as well as improved HIV prevention and control measures. More targeted and relevant HIV prevention programs for older and less educated MSM are urgently needed to address the key risk factors we have identified. MSM engaging in drug-related risk behaviours require multi-strategy HIV interventions relating to both sex and drug behaviour among MSM and their partners who engage in drug use. Further work is needed to identify locations and strategies where these high-risk individuals can be accessed as well as to reduce barriers related to social discrimination and stigma. Targeting high risk individuals and groups should supplement existing efforts aimed at the MSM population in HCMC.

## Background

Globally, the HIV epidemic appears to have stabilised in recent years and the annual number of new HIV infections has slightly declined [1, 2]. However, the epidemic picture varies greatly in different regions and remains critical in South-East Asia with 4.8 million people living with HIV/AIDS (PLWH) in 2010. Vietnam, together with six other countries in Asia, namely India, China, Thailand, Indonesia, Malaysia and Myanmar contributed over 90 % of the total number of PLWH in the region in 2009 [3].

HIV has spread across Vietnam since the first case was identified in 1990 [4, 5]. The number of PLWH in Vietnam was estimated to rise from 156,802 in 2009 to 256,000 in 2014 (accounting for 0.26 % of the general population) [6, 7]. Although the number of HIV newly reported cases has decreased in Vietnam (roughly 14,000 new cases per year from 2010 to 2013), a growing concern about the HIV epidemic is the very high prevalence of HIV among sub-populations such as intravenous drug users (IDU), MSM and female sex workers (FSW) [8–10]. There have been significant falls in HIV prevalence among IDU from 29.35 % in 2002 to 22 % in 2013 and among FSW from 5.9 % in 2002 to 5.3 % in 2013 [8, 11, 12]. However, there are signs of an increase in HIV prevalence among MSM. HIV infection among MSM was projected to increase from 1.7 % in 2005 to 2.4 % in 2013, but a recent HIV sentinel survey showed a significant increase from 2.26 % in 2012 to 3.69 % 2013 [8]. This trend was even more serious in the two largest cities, HCMC and Hanoi. Statistics showed a growing HIV epidemic among MSM in these two cities with prevalence changing from 5.3 % and 9.4 % in 2006, to 14 % and 20 % in 2009 respectively [13–15]. In recent years, with rapid social and cultural changes, a growing number of men have openly identified themselves as MSM in Vietnam [4]. Further, recent studies show that the MSM populations are largely concentrated in urban areas especially in HCMC and Hanoi [14, 15]. The proportion of the MSM population in these two cities is much larger, with 3 % of total male population over 15 years old, compared with only 1.5 % in other cities/provinces [11, 14]. HCMC is the biggest city in Vietnam and the major “hot spot” of the HIV/AIDS epidemic, with a prevalence of about 2 %, accounting for 36.5 % of the total national HIV/AIDS patients [16, 17]. The annual number of new HIV infections among MSM is projected to be higher than the new infections among IDU and FSW in HCMC [15].

While a wide range of studies and reports related to the HIV/AIDS epidemic is available for IDU and FSW in Vietnam, there are few studies, intervention programs and reports published on the MSM population [4, 11, 13, 16–19]. Although MSM are now a target group for HIV surveillance in Vietnam, previous exclusion of MSM in national surveillance, together with limited awareness of the risk of HIV transmission, inadequate knowledge and understanding about the disease and its prevention among MSM, may have contributed to the high level of HIV infection in this group [15–17, 20]. Further, stigma and discrimination against homosexuality at multiple levels (in families, local communities, legally and at law enforcement levels) may drive this population socially ‘underground’ and conceal related HIV infection, making it more difficult for MSM to access HIV testing, prevention and treatment services [21–23].

Although the proportion of MSM across all age groups may be similar, recent studies describing the socio-demographic characteristics of MSM, found that the MSM population in Vietnam is young (79 % aged 20-39), single, with low income [4, 15, 17], low education (mean level of education at grade eight) and having a wide range of employment [17]. In terms of their self-identified sexuality, studies show that MSM classified themselves as “Bong lo”, openly gay men, or “Bóng kín” which refers to MSM who conceal their homosexuality and may live a heterosexual lifestyle. Less than 15 % of the participants in previous studies identified themselves as being in the “Bong lo” group [17, 24–26]. Some MSM reported living with a female partner or having a wife and children (3.4–8.7 %) [4, 17, 24–26]. International literature also indicates that MSM engaging in high-risk behaviours with limited knowledge about HIV have contributed substantially to HIV infection in this group [4, 15, 17]. Behaviour risk factors identified by MSM studies included lack of condom use, involvement in commercial sex, and drug use. HIV prevalence among bisexual MSM has been found to be twice as high as that in homosexual groups, and having multiple sex partners or group sex increased the risk of HIV infection in Vietnam [17, 26]. Anal sexual intercourse (ASI) (including unprotected anal sexual intercourse (UASI)) with a male partner is a risk factor for HIV transmission. In addition, low condom and lubricant use are also common risk factors [13, 17, 27, 28]. Injecting drugs is identified as another major risk for HIV infection among MSM [29]. Less than one in five MSM in HCMC appeared to have adequate knowledge about HIV transmission or prevention, although approximately half of them believed that homosexual men were more likely to be infected with HIV than other men [13, 17, 30].

Such factors have put MSM at higher risk of HIV infection in Vietnam. Despite the risk factors identified by previous studies and reported above, the percentage of MSM ever accessing HIV testing, HIV prevention programs, care/treatment and support remains low. National data indicate a growing epidemic among MSM particularly in HCMC, with HIV prevalence projected to reach 16 % in 2013 [8, 31].

The lack of large-scale epidemiological studies conducted on MSM in Vietnam has meant that understanding the socio-demographic and behavioural risk factors of HIV infection in this population remains limited [4, 17, 25, 32]. To monitor HIV prevalence and inform policy development for HIV control and prevention, the Vietnamese government has been working with WHO and UNAIDS to establish an Integrated Biological and Behavioural Surveillance (IBBS) system since the 1990s with recent data collections conducted in three rounds: 2006, 2009 and 2013 (data not yet available) [8, 13, 14]. Data collected through the surveillance system, particularly targeting high-risk subgroups including MSM and their behavioural risks, are considered high quality and also serve as a reference for international best practice. Global HIV/AIDS research emphasises the importance of addressing multiple risk factors related to the behaviours among such high-risk subgroups, to improve control of the epidemic and to prevent transmission to the general population [1, 33].

This study used data from the IBBS Round II to identify and understand socio-demographic characteristics, risk behaviours, knowledge levels and access to preventive programs related to HIV infection among MSM. These data enable a measurement of prevalence among MSM population in HCMC that can pave the way for more effective interventions and health education programs to prevent HIV/AIDS and other sexual transmitted infections (STIs) for this group both because it is important in its own right but also because it is a potential “bridge” to the general population.

## Methods

This project was based on a secondary analysis of the IBBS data collected in Vietnam in 2009–2010 to examine the research question: What are the socio-demographic and behavioural risk factors contributing to HIV infection among the MSM population in HCMC? A cross-sectional design was employed to sample males aged over 15, who reported having any types of sex (oral, anal or mutual masturbation) with another man at least once during the prior 12 months, from communities in HCMC. The participants were recruited using the respondent driven sampling (RDS) methodology. RDS involves asking individuals to refer people in their communities or social networks to the survey and is often chosen to obtain probability samples from hard-to-reach populations that are highly stigmatized and therefore are largely hidden [34]. It also enabled this study to recruit a representative sample of MSM in HCMC. The primary participants “seeds” were chosen by the local staff at data collection centres to ensure a broad diversity of socio-demographic characteristics and geographic areas of MSM networks. Each seed was given three referral coupons to invite their peers to participate. The subsequent participants were also required to invite additional peers within their networks. A small incentive (approximately USD$3–6) was offered to each participant at the time of interview and an additional incentive was provided to those who recruited their peers to participate in the study. The RDS process continued for five to eight rounds of referrals until the target sample size (*n* = 400) was reached.

The sample consisted of 399 MSM who were residing or working in four districts in HCMC from June 2009 to February 2010. The IBBS data included biological data (specimens) and behavioural data collected through a questionnaire survey. All data were collected at study centres which were set up on the basis of geographic convenience and accessibility for participants. The dependent variable, HIV infection, was determined by biological data; and the independent variables including socio-demographic characteristics, risk behaviours, sexual practices and risk perception for HIV infection were collected by a questionnaire administered by trained interviewers. The IBBS protocol, questionnaires and consent forms and other tools were jointly approved by Vietnam Ministry of Health (MOH), the Ethics Review Board of the National Institute of Hygiene and Epidemiology (NIHE), the FHI (Family Health International) Protection of Human Subjects Committee and the U. S. Centre for Disease Control and Prevention (CDC) Internal Review Board. Permission to use these data was granted by the Vietnam Administration of HIV/AIDS Control (VAAC) - Vietnam Ministry of Health, NIHE and FHI which are the leading organizations involved in conducting this survey. Participation in the study was completely voluntary and strictly anonymous. Complete confidentiality of the participant data was emphasised.

Original data were entered in STATA database format; this was then converted to SPSS version 20.0 which was used for analysis in this research project. Descriptive statistics were first employed to describe socio-demographic characteristics of the whole sample and then univariate analysis was used to explore the unadjusted (crude) association of the dependent variable (HIV infection) and each independent variable (demographic, knowledge and risk behaviours). To examine the effect of demographic variables together with other risk factors on the dependent variable (HIV infection), multiple logistic regression was applied. All independent variables which were identified as significant variables regarding their associations with HIV infection in univariate analysis and in the literature review were included in the logistic regression modelling to examine their inter-relationships and overall effect on the participants’ HIV outcomes. A *p*-value of less than 0.05 was considered as evidence of a significant association. A 95 % confidence interval for each independent variable was also obtained to estimate the risk (using adjusted odds ratio, OR) between the two subgroups under each independent variable.

## Results

Of the 397 participants for whom an HIV test was obtained (2 had missing data) 14.8 % (*n* = 59) were found to be HIV(+). The distribution of socio-demographic characteristics among the total participants in relation to HIV outcomes are summarised in Table 1. Overall, the mean age of the MSM sample was 26.8 years at the time of interview (Std. Deviation (SD) =8.1) with 53.9 % of the sample aged 25 years or younger. The majority of the MSM participants (63.7 %) completed only primary or secondary education. The proportion of unemployed participants among MSM population was high at 21.8 %. The median income of the participants per month was 1.8 million VND (USD$86). About 65 % of participants had a monthly income of less than 2 million VND (USD$96).

Over two thirds (68.4 %) of the MSM sample identified themselves as “Bóng kín” and nearly a third (28.8 %) identified themselves as being straight. Only a small percentage (2.8 %) reported themselves as “Bong lo”. At the time of interview, the proportion of the MSM sample who had ever married a woman was 11 % (*n* = 44).

Descriptive analyses were used to present the distributions of socio-demographic characteristics in relation to HIV positive and negative outcomes. Univariate analyses (including chi-square tests and t tests) were used to identify the associated socio-demographic variables, behavioural risk factors and knowledge about HIV and STI with HIV infection (the outcome). About 54 % of the MSM sample were 25 years old or younger and 75 % of them were classified in the low and medium income groups. The majority of the sample (68.4 %) identified themselves as “Bóng kín”, while only 28.8 % considered themselves as “straight”. Almost a half of the participants (48.9 %) preferred to have sex with men only, but the proportion of bisexual men (47.9 %) was at a similar level. Among the socio-demographic variables, age, education and income (*p* < 0.001, *p* = 0.005 and *p* = 0.004 respectively) showed significant associations with HIV infection in the results (Table 1).

The behavioural risk factors (35 items), knowledge about HIV and STI (10 items) and access to preventive services (5 items) that were significantly associated with HIV infection included having anal sex with male partners in the last month, having STIs, visiting an STI clinic for STI screening in the last 12 months, ever injecting drugs, having sexual partners who injected drugs, feeling at risk of HIV infection, ever receiving an HIV test and not receiving free lubricant in the last 6 months (all *p* values <0.05; data not shown). Among all MSM participants, more than 70 % had anal sexual intercourse (ASI) and almost 85 % of the HIV (+) MSM had ASI with their male partners in the last month. Also, about a quarter (25.3 %) of the sample reported that they had used drugs previously in their lives and 8.1 % reported that they had injected recreational drugs. In addition, 30.8 % of all MSM and 47.5 % HIV (+) MSM reported having partners who injected drugs. Only 16.9 % of the HIV (+) MSM had visited an STI clinic for STI screening in the last 12 months, while the percentage among HIV (-) participants was almost double (30.8 %). Over one fifth (21.3 %) of the participants had laboratory confirmed STIs and 20.3 % HIV (+) MSM had STI coinfections. Of all participants, 44.9 % perceived they were at high risk of HIV infection but only about one quarter (25.6 %) had ever had tested for HIV. It is also noted that a much higher percentage of HIV (-) participants had ever received free lubricant than those who were HIV (+) (29.0 % vs. 8.5 %). Statistical tests did not find significant associations between the remaining items under ‘behavioural risk factors’ and ‘HIV/STI knowledge and services’ categories. Although a slightly higher proportion of *‘never/sometimes used condom during anal sex*’ was found in HIV (+) (54.2 %) participants compared with HIV (-) MSM (44.7 %), the association with HIV infection was not significant (data not shown).

Socio-demographic and behavioural risk factors with significant associations with HIV prevalence in the univariate analyses, together with ever selling sex, having sex with sex workers and using drugs in the last 12 months, which were identified as common risk factors in previous studies, were included in multiple logistic regression analysis. The model was developed to identify the significant variables in predicting HIV outcomes and to determine the strength of associations (adjusted odds ratios, OR) between the significant factors and HIV infection among the MSM participants. From the multivariate analysis shown in Table 2, two demographic factors were found to be significantly associated with HIV infection: age group and educational level of the participants. In this model, the MSM aged over 25 were more likely to be HIV (+) than the younger group (OR = 7.82, 95 % CI = 3.37–18.16, *p* < 0.001). Further, participants who had low educational levels (OR = 2.74, *p* < 0.05) and medium education levels (OR = 2.68, *p* < 0.05) were more likely to be HIV (+) than those with a higher educational level.

**Table 1:** Univariate analysis results between the socio-demographic characteristics and HIV infection

Variables	HIV Infection status				
	Total N (%)	HIV(+)	HIV(-)	Unadjusted OR/(95%CI)^c^	p- value^d^
Total	397	59 (14.8)	338 (84.7)		
Age				Mean difference	
Mean (SD)	26.8 (8.1)	30.0 (6.5)	26.3 (8.3)	3.31 (1.80-5.60)	<0.001***^a^
Age Group (n)					
Over 25	183 (46.1)	46 (78.0)	137 (40.5)	5.19 (2.70-9.97)	<0.001***
25 or less	214 (53.9)	13 (22.0)	201(59.5)	1	
Monthly income (VND million)^b^					
Mean (SD)	2.05 (1.2)	1.7 (0.9)	2.1 (1.4)	-0.86 (-0.68 to -0.12)ǂ	0.005**
Median	1.8				
Range	0.1 – 8.5				
Income group per month					
Low (≤1.0VND million)	101 (25.7)	18 (30.5)	81 (24.0)	1.69 (0.82-3.52)	0.331
Medium (1.0-2.0)	154 (39.6)	25 (42.4)	129 (38.2)	1.48 (0.75-2.90)	
High (>2.0)	138 (35.1)	16 (27.1)	122 (36.1)	1	
Education Level (n)					
Low (Under grade 5)	95 (23.9)	19 (32.2)	75 (22.2)	3.40 (1.50-7.68)	0.004**
Medium (Grade 6-9)	159 (39.8)	30 (50.8)	129 (38.2)	3.12 (1.46-6.63)	
High (Grade 10 or over)	145 (36.3)	10 (16.9)	134 (39.6)	1	
Employment status(n)					
Unemployed	85 (21.8)	17 (28.8)	68 (20.1)	1.61 (0.86 – 3.00)	0.133
Employed	312 (78.2)	42 (71.2)	270 (79.9)	1	
Self-identification					
Straight	115 (28.8)	20 (33.9)	95 (28.1)	1	0.600
Bóng kín	273 (68.4)	38 (64.4)	233 (68.9)	0.77 (0.43-1.40)	
Bong lo (homosexual)	11 (2.8)	1 (1.7)	10 (3.0)	0.48 (0.06-3.920	
Sexual orientation					
Bisexual	191 (47.9)	31 (52.5)	160 (47.4)	1	0.276
Heterosexual	13 (3.2)	0 (0)	13 (3.8)	-	
Homosexual	195 (48.9)	28 (47.5)	165 (48.8)	0.88 (0.50-1.52)	
Ever married a woman					
Yes	44 (11.0)	8 (13.6)	36 (10.7)	1.32 (0.58-2.99)	0.206
No	355 (89.0)	51 (86.4)	302 (89.3)	1	

^a^Comparing mean difference between HIV(+) and HIV(-) using independent sample t test

^b^1USD ≈ 18,000 - 20,000VND at the end of 2009 and beginning of 2010 (at interview time)

^c^95%CI: 95% confidence interval

^d^* P<0.05; ** P<0.01; ***P<0.001 (2-tailed tests).

**Table 2:** Multivariate logistic regression analysis for the relation between the factors associated with HIV infection among MSM in HCMC

Variable	N (%)	OR	95%CI	P value^a^
Age group				
Over 25	184 (46.1)	7.82	3.37-18.16	<0.001***
25 or less	215 (53.9)	1		
Income group per month?				
Low define these?	101 (25.7)	0.91	0.40-2.04	0.811
Medium	154 (39.6)	1.2	0.46 - 3.11	0.709
High	138 (35.1)	1		
Educational level				
Low (Under grade 5)	95 (23.9)	2.74	1.10-6.83	0.030*
Medium (Grade 6-9)	159 (39.8)	2.68	1.00-7.14	0.049
High (Grade 10 or over)	145 (36.3)	1		
Employment status				
Unemployed	87 (21.8)	1.54	0.63-3.79	0.343
Employed	312 (78.2)	1		
Self-identification				
Straight	115 (28.8)	5.48	0.47-64.41	0.176
Bong kin	273 (68.4)	2.86	0.28-29.06	0.375
Bong lo	11 (2.8)	1		
Sexual orientation				
Bisexual	191 (47.9)	1.1	0.47-2.61	0.824
Heterosexual	13 (3.2)	N/A		0.998
Homosexual	195 (48.9)	1		
Ever married a woman?				
Yes	44 (11.0)	1.02	0.38-2.85	0.965
No	355 (89.0)	1		
Having anal sex with male partners in past month				
Yes	292 (73.2)	2.7	1.10-6.64	0.030*
No	107 (26.8)	1		
Selling sex				
Yes	216 (54.1)	1.07	0.54-2.13	0.841
No	183 (45.9)	1		
Having sex with SWs				
Yes	72 (18.0)	1.08	0.46-2.53	0.855
No	327 (82.0)	1		
STI infection				
Yes	85 (21.3)	2.16	0.92-5.07	0.078
No	309 (77.4)	1		
Ever used drugs				
Yes	101 (25.3)	1.37	0.55-3.43	0.503
No	298 (74.7)	1		
Ever injected drugs				
Yes	32 (31.4)	0.89	0.25-3.15	0.759
No	70 (68.6)	1		
Have sexual partners who injected drugs in past 12 months				
Yes	123 (30.8)	2.24	1.06-4.73	0.035*
No/Don’t know	276 (69.2)	1		
Feel risk of HIV infection				
Yes	175 (44.9)	2.42	1.24-4.73	0.006*
No/Don’t know	215 (55.1)	1		
Ever had HIV test				
No	288 (72.2)	2.46	0.89-6.83	0.084
Yes	102 (25.6)	1		
Visit STI clinics without symptoms				
No	285 (71.1)	1.38	0.56-3.42	0.483
Yes	114 (28.6)	1		
Received free lubricant in past 6 months				
Yes	103 (25.8)	2.62	0.83-8.32	0.101
No	296 (74.2)	1		

^a^* P<0.05; ** P<0.01; ***P<0.001 (2-tailed tests)

Multivariate analysis revealed that having anal sex with male partners was also a strong predictor of HIV infection (OR = 2.7, *p* < 0.05). Similarly, having sexual partners who injected drugs during the past 12 months increased the likelihood of HIV infection (OR = 2.24, *p* = 0.035). MSM who were HIV (+) were more likely to feel themselves at risk of HIV infection than the HIV (-) MSM (OR = 2.42, *p* = 0.006). It was noted that STI infection and ever receiving an HIV test were significantly associated with HIV infection in the univariate analyses but the associations were only at the borderline of significance in the logistic regression model (*p* = 0.078 and 0.084 respectively).

## Discussion

This study found that the prevalence of HIV infection among MSM in HCMC was 14.8 % in 2009. Evidence indicates that HIV prevalence increased among the MSM population in HCMC from 5.3 % in IBBS Round I (2006) to 14.8 % in IBBS Round II (2009) [13–15, 27]. This is one of the highest levels of HIV prevalence found in studies of MSM in South East Asia, following Mandalay in Myanmar (about 22.0 % in 2010) [35] and Bangkok in Thailand (24.7 % in 2010) [36]. However, there has been a decline in HIV prevalence in both Mandalay (from 35 % in 2007 to 22.0 % in 2010) [35] and Bangkok (from 30.8 % in 2007 to 24.7 % in 2010) [36, 37], while the rate has nearly tripled in HCMC. These data support a recently published systematic review of over a dozen studies of MSM in Vietnam that identified a rapidly increasing HIV (+) rate among this high-risk population over the last decade [15].

Regarding the socio-demographic characteristics of the MSM sample in this study, the mean age of the MSM participants was 26.8 years, and approximately two thirds had a low to medium income (65.3 %) and over 60 % (63.7 %) of participants had low to medium education levels. However, the HIV (+) MSM were more likely to be over 25 years than the HIV (-) group (OR = 7.82, *p* < 0.001). It is likely that as MSM get older they will have more chance of having been infected over time and therefore prevalence within older age will be higher than in the younger groups. Hence, this finding is not unexpected. This demographic profile of the MSM sample with older age and relatively low education levels having higher levels of infection could be associated with less effective HIV prevention and communication strategies during the early years of the epidemic. It is similar to the HIV (+) MSM in neighbouring countries such as China [38, 39] and Thailand [40]. Multivariate analysis indicated that MSM with low or medium educational levels were significantly associated with HIV infection. This supported results from previous studies in Vietnam showing that many older MSM had lower education than younger MSM and were more likely to be married and had sex with both men and women. Therefore, this subgroup has the potential to transmit HIV beyond the MSM network [17]. A study conducted in four cities in China presented a similar result, showing that HIV (+) MSM were 80 % more likely to have a relatively low education [38]. The 2006 IBBS study in Vietnam (Round I) showed that 52.7 % MSM had completed high school or tertiary education [13], whereas in this study (IBBS Round II, 2009), only 36.3 % had attained this educational level. Between 2006 and 2009 there was a substantial growth in the proportion of MSM with lower educational levels at a higher risk of HIV infection in HCMC.

In 2009, 11 % of MSM participants were, or had been married. This proportion was much higher than the results from IBBS in 2006 (5.0 %) as well as other studies in Vietnam indicating a range from 3.4 % to 8.7 % [13, 17]. This proportion was also at the higher end of the scale that ranges from 3 % to 13 % among South East Asian countries [41]. The increased number of married MSM is of concern as they may provide a bridge for STI and HIV transmission to the heterosexual population [17]. The percentage of MSM who preferred having sex with men was 48.9 %, although the percentage of MSM who preferred having sex with both men and women (47.9 %) was higher than that reported from previous studies (range 9.2–47 %) [2, 42] and from other countries, such as Thailand (28.8 %) [40]. The majority of MSM (68.4 %) still hid their sexual orientation, compared to 54.4 % in 2006, and only 2.8 % MSM were “out” or publicly homosexual, which was much lower than in 2006 (10.8 %) [13].

Vietnam has sensitive cultural and social norms. Men who engage in homosexual behaviours are subject to social stigma and discrimination [20]. The results from a qualitative study [32] indicated that getting married to a woman was perceived as the best way to avoid stigma and discrimination from families and communities. As a result, most MSM tend to hide their homosexual behaviour, while others decide to marry women to satisfy their families and to avoid social stigma from their families and communities [22, 32, 42]. The high percentage (68.4 %) of “Bóng kín” MSM together with a considerable proportion of married (11.0 %) and bisexual (47.9 %) MSM suggests the potential for such MSM to play a role as a bridging population which could contribute to the transmission of HIV/STDs to women, especially by men who engage in high risk behaviours such as IDU and MSM sex workers [8]. Although they may have access to healthcare, such care may well be less effective if “Bóng kín” MSM cannot be open about their sexuality due to stigmatisation and discrimination toward homosexuality [8, 21, 23]. A further outcome of such stigmatisation and social discrimination may be restricted access to HIV preventive programs and interventions. Despite the lack of association between marital status, sexual orientation and self-identity with HIV infection in this study, the risk of HIV transmission through married, bisexual and “Bóng kín” MSM to their homosexual and bisexual partners would benefit from further research to identify strategies and opportunities to disseminate safe sex messages and condoms/lubricants.

The “Bong lo” group, having come ‘out of the closet’, is more likely to face stigma and discrimination from their families and communities and face more discrimination and difficulties in life than the “Bóng kín” group. There is evidence to show that this may have limited their opportunities and motivation for tests for HIV and receiving counselling [31, 32].

In the literature, MSM were reported as having multiple risk behaviours such as trading sex, having unprotected ASI, having multiple sexual partners including female partners and drug use [8, 15, 17]. Our results showed that the HIV (+) MSM were more likely to have had ASI with male partners during the past 1 month than the HIV (-) MSM. This result was consistent with a study in Thailand where HIV (+) MSM were likely to engage in ASI [40] and evidence from China indicating that having ASI was significantly associated with HIV infection [43].

In terms of drug use, 25.3 % of the MSM sample reported ever using recreational drugs and 8.1 % said they had previously injected recreational drugs, which is higher than figures from previous studies in Vietnam (range 3–22.8 % for drug use and 1.5–3.8 % for injecting drugs) [17, 40]. Of MSM who ever used drugs, approximately one third (31.4 %) had ever injected drugs which was about 10 % higher than in the 2006 IBBS survey [13]. Despite the higher percentages of using and injecting drugs among MSM, there was no association found between drug use or injecting drugs and HIV infection in the multivariate analyses in our research. This finding was different from the results of several studies that showed that drug use played a significant role in HIV infection among MSM [17, 44, 45]. However, having sexual partners who injected drugs was highly associated with HIV infection among MSM (OR = 2.24, *p* < 0.05). Three in ten MSM in our study reported having partners who injected drugs, this proportion was triple the result found in 2006 IBBS (10.3 %) [13]. Further studies are necessary to investigate the relationship between substance use and HIV infection among MSM and their partners in order to improve our understanding of the relationships between these variables and pathways of HIV infection.

Generally, STI infection was high (21.3 %) among the MSM participants. STI was found to be a potential predictor for HIV infection among the MSM sample in HCMC in this study. HIV (+) MSM were more likely to consider themselves at risk of HIV infection than HIV (-) MSM (OR = 2.42, *p* < 0.05). In comparison with the 2006 IBBS study, a higher proportion of MSM felt at risk of HIV infection in the 2009 survey (44.9 % in 2009 vs. 30.1 % in 2006) [13]. Despite this, only a quarter (25.6 %) of MSM had ever had an HIV test in this study. Future studies could address this issue to develop a more complete understanding with a possible explanation of their behaviours, in order to develop more targeted HIV prevention interventions for the MSM population.

### Limitations of the study

As the IBBS data were collected from a cross-sectional survey, it is not possible to demonstrate a causal relationship between the risk factors and the outcome among the MSM population in HCMC. Also, IBBS data from the questionnaire used were self-reported including data on sexual behaviours, drug use or injecting drug history, which are still sensitive issues in Vietnam. Therefore, some information bias (such as under reporting, recall bias, non-disclosure and social desirability bias) may have occurred and this may have affected the estimates of the strength of associations between the factors and the outcome. In addition, the actual proportion of refusal cannot be evaluated using RDS; and some respondents received more than one invitation coupon from multiple recruiters due to the overlapping social networks/ peer groups among the seed participants (however they had participated only once). Some potential non-response bias, as well as “seeds selection” bias which might require RDS adjustment could still have occurred. Some adjustments were made through reducing the referral coupons to one and subsequently to zero in order to balance the sizes of different seeds networks of MSM as the participant number was close the target sample size. However, there are potential issues concerning selection bias as some MSM networks may remain underrepresented.

## Conclusion

As MSM in Vietnam have a high HIV prevalence and there is an increasing population of MSM in HCMC, there is a need to improve our understanding of their patterns of behaviour, risk practices and social networks and connections in order to develop more effective and targeted HIV control measures. This study found high HIV prevalence among the MSM participants in HCMC (14.8 %). Older MSM, low educational levels, having anal sex with male partners, having sexual partners who inject drugs and self-reported feeling at risk of HIV infection were all associated with HIV infection among the MSM population in HCMC.

Due to the high proportion of MSM engaging in drug-related risk behaviours, multi-strategy HIV interventions for MSM and their partners who engage in drug use should be supported to reduce risk. Peer support, additional targeted services such as IDU education and information sessions provided to MSM and associated support groups, needle exchange opportunities should be investigated. More targeted and relevant HIV prevention programs are urgently needed to address the key risk factors identified above. Also, further work is needed to reduce barriers related to social discrimination and stigma, supplementing existing efforts aimed at the MSM population in HCMC. Further studies should be conducted within this hidden population in order to provide a better understanding of the context of MSM in Vietnam and to prevent the potential expansion of HIV transmission among MSM in particular and, more broadly, to the general population.

### Ethics and consent to participate

This project was based on a secondary analysis of IBBS data collected in Vietnam in 2009–2010. The study team obtained permission from Vietnamese Ministry of Health for the use and analysis of the IBBS data. In Australia, studies using secondary analysis do not require ethics approval.

### Consent to publish

Not applicable. This manuscript does not contain any individual person’s data in any form. All participants were de-identified in the dataset.

### Availability of data and materials

The data used in this manuscript are not publicly available but can be obtained upon request to the first author.

## Abbreviations

ASI: anal sexual intercourse
FHI: Family Health International
FSWs: female sex workers
HCMC: Ho Chi Minh City
RDS: respondent driven sampling
IDUs: intravenous drug users
MSM: men who have sex with men
NIHE: National Institute of Hygiene and Epidemiology, Vietnam
PLWH: people living with HIV
STIs: sexually transmitted infections
